# Maternal Deprivation Increased Vulnerability to Depression in Adult Rats Through DRD2 Promoter Methylation in the Ventral Tegmental Area

**DOI:** 10.3389/fpsyt.2022.827667

**Published:** 2022-03-02

**Authors:** Zhenli Guo, Shansi Li, Jialing Wu, Xiongzhao Zhu, Yi Zhang

**Affiliations:** ^1^Medical Psychological Center, The Second Xiangya Hospital, Central South University, Changsha, China; ^2^Medical Psychological Institute of Central South University, Central South University, Changsha, China; ^3^National Clinical Research Center for Mental Disorders, The Second Xiangya Hospital, Central South University, Changsha, China

**Keywords:** maternal deprivation, depression, DRD2, DNA methylation, ventral tegmental area

## Abstract

**Objective:**

Early life adversity is a risk factor for depression in adulthood; however, the underlying mechanisms are not well understood. This study aims to investigate the effect of DNA methylation of DRD2 gene on early life stress–induced depression in adult rats.

**Methods:**

Newborn Sprague–Dawley rats were randomly assigned to four groups: maternal deprivation group (MD), chronic unpredictable stress (CUS) group, maternal deprivation plus chronic unpredictable stress (MD/CUS) group, and normal control group (NOR). Behaviors were measured by open field test (OFT), sucrose preference test (SPT), and Original Research Article forced swimming test (FST). Fecal CORT level was detected by ELISA. Bisulfite amplicon sequencing PCR was used to assess methylation levels of DRD2 promoter.

**Results:**

CUS and MD/CUS rats had a significantly shorter total distance, longer immobility time, and higher CORT level, while MD and MD/CUS rats had a significantly lower percentage of central distance, more feces, lower rate of sucrose preference, and lower levels of DRD2 protein and mRNA in the VTA than NOR rats. CUS rats showed a significantly higher DRD2 mRNA and protein levels in the VTA than NOR rats. CUS, MD, and MD/CUS rats showed a significantly higher level of DRD2 promoter methylation than NOR rats. CORT level was significantly correlated with the sucrose preference rate in SPT, the immobility time in FST, the total distance, and the number of fecal pellets in OFT. DRD2 protein level was significantly correlated with the sucrose preference rate and the number of fecal pellets. DRD2 mRNA level was significantly correlated with the percentage of central distance and the number of fecal pellets in OFT. The level of DRD2 promoter methylation was significantly correlated with the sucrose preference rate, immobility time, total distance, the percentage of central distance, and the number of fecal pellets.

**Conclusions:**

Early life MD increased vulnerability to stress-induced depressive-like behavior in adult rats. Enhanced DRD2 promoter methylation in the VTA may increase the susceptibility to depression.

## Introduction

Depressive disorder is one of the most prevalent psychiatric illnesses. Data from WHO demonstrated that the lifetime prevalence of depression is as high as 18.1% globally (1). The etiology of depression is complex with risk factors that can enhance the vulnerability being a family history of depression (2) and childhood traumatic experiences (3). The childhood or early life period is particularly sensitive to adverse events that increase the risk of depression (3, 4). However, this predisposition, or vulnerability to depression is poorly understood.

Dopamine (DA) is the most abundant monoamine neurotransmitter in the CNS and regulates motivation, emotions, reward circuits, cognition, and reinforcement behaviors (5, 6). It has been widely reported that dopaminergic neurotransmitter is deficient in depressive individuals (7, 8). Mesocorticolimbic DA circuitry, originating from the ventral tegmental area (VTA) neurons, is a key part of the brain's reward circuitry and plays an important role in mediating stress response. The VTA DA neurons have been suggested to be highly susceptible to stress and are involved in the pathophysiology of stress-induced depression (9, 10). The dopamine D2 receptor (DRD2) is one of the major DA receptor subtypes. In the VTA dopaminergic neurons, DRD2 expression in pre-synaptic (autoreceptors) can inhibit the excitability of dopaminergic neurons by regulating firing rate and plays a negative feedback regulation on dopamine neurons (11). Previous studies show that DRD2 plays a key role on the development of stress-related depression (11). The study of Chen et al. (12) found that CUS induces depression-like behaviors and upregulates the expression of DRD2 in the prefrontal cortex of rats. Depression-like behaviors were observed more pronounced in DRD2 knockout (DRD2^−/−^) mice than wild-type mice following chronic stress (13). Our previous study also revealed that early life maternal deprivation (MD) induces aberrant expression of DRD2 gene in the mesocorticolimbic DA system of adult rats, especially elevates DRD2 gene expression in the striatum, and reduces DRD2 gene expression in the VTA (14–17). However, the molecular mechanisms regulating DRD2 gene expression have yet to be fully understood.

DNA methylation has been demonstrated to be a stable epigenetic imprint after stress and can consistently repress gene expression. DNA methylation in mammals currently occurs mainly on cytosines in the CpG islands of the gene promoter region. Hypermethylation in the promoter region can spatially prevent DNA from binding to transcription factor complexes and repress gene expression, while demethylation restores it (18). DNA methylation can be affected by early life stress with a long-lasting change, which can alter disease susceptibility later in life, including depression (19–21). For example, Tozzi et al. (22) found that childhood maltreatment can induce demethylation of the FK506 binding protein 5 (FKBP5, a regulator of the glucocorticoid receptor) may be associated with clinical symptoms of depression disorder. McGowan et al.'s (23) study in the postmortem of suicide victims found hypermethylation in the neuron-specific glucocorticoid receptor promoter in hippocampus of victims with early life adversity compared with that without early life adversity. It has been shown that methylation of the dopamine D2 receptor (DRD2) gene promoter is elevated in women who were abused during childhood (24). However, it should be further clarified that early life stress may enhance vulnerability to depression through changing DNA methylation of DRD2 gene in adulthood.

In this study, maternal deprivation (MD) and chronic unpredictable stress (CUS) were used, as early life stress and adulthood stress paradigms, respectively, to establish animal models of depression. The level of DNA methylation and expression of DRD2 gene in the VTA were investigated.

## Materials and Methods

### Animals and Design

Pregnant Sprague–Dawley (SD) rats (SLAC Laboratory Animal Inc., Shanghai, China) were housed in the Experimental Animal Center of the Second Xiangya Hospital, Central South University and checked daily for delivery. The date of birth was designated as postnatal day 0 (PND 0). On PND 1, litters were randomly divided into four groups: MD, CUS, MD plus CUS (MD/CUS), and normal control (NOR). Rats in MD and MD/CUS group were exposed to MD stress from PND 1 to PND 14. Rats in CUS and MD/CUS group were exposed to CUS for 28 days at the age of 10 weeks; the NOR rats received no MD and CUS. Only male rats were included and there were 10 rats in each group. Forty male rats were weaned on PND 21 and housed in separate cages (two rats/cage). The behaviors of all male rats were assessed at 14 weeks old and 24-h fecal corticosterone (CORT) was collected before behavioral tests. All male rats were decapitated within 24 h after the end of the behavioral tests and brain tissues were taken for biological experiments. The animal protocol was approved by the Animal Ethics Committee of the Second Xiangya Hospital, Central South University. During the experiment, the rats can drink and eat freely with a light and dark cycle of 12 h/12 h (lights on 8:00–20:00), room temperature of 21–23°C, and humidity of 50–55%.

### Maternal Deprivation (MD)

The MD paradigm was performed according to a previous publication (25). The litters in MD and MD/CUS groups were moved from the dams to a single standard polycarbonate box (26 × 20 × 14 cm), paved the bottom of the box with wood chips at 9:00, and then moved back the dams at 15:00 from PND 1 to PND 14 for MD. Rats were weaned at PND 21 and housed in separate cages for every two rats until the 10th week.

### Chronic Unpredictable Stress (CUS)

The CUS paradigm was established following a published protocol with a small modification (26). The protocol included seven stressors: crowding (5–6 rats/cage, 2 h), wet bedding (15 h), elevated open platform (10 × 10 cm, 160 cm in height, 2 h), restraint (2 h), water deprivation (16 h), food deprivation (16 h), and blank stimulus. CUS and MD/CUS rats received a stressor every day from 10 to 14 weeks old. The same stressor cannot occur for two consecutive days so that the rats could not predict the occurrence of the stressor.

### Open Field Test (OFT)

The OFT was used to assess locomotor activity and anxiety-like behavior. All rats were tested and sequentially placed in the very center of a box (50 × 83 × 56 cm). A video camera was used to record activities of rats in the box for 5 min. The total distance (index for locomotor activity), vertical counts (index for exploration), the percentage of central distance, and the number of fecal pellets (both indexes for anxiety level) were automatically recorded with a computerized tracking system (Ethovision 1.50; Noldus IT, Wageningen, Netherlands). The percentage of central distance represented the percentage of central distance to the total distance during the test. The box was fully cleaned with ethanol (75%) after each trial.

### Sucrose Preference Test (SPT)

The SPT was used to assess anhedonia of rodents and performed by a published protocol (26). The protocol included three procedures: (1) rats received two bottles of 1.5% sucrose solution for 24 h; (2) rats received one bottle of 1.5% sucrose solution and one bottle of plain water for the next 24 h; (3) rats were deprived of water and food for the next 18 h. Two pre-weighed bottles of solution (respectively 1.5% sucrose solution and plain water) were then provided to rats for 1 h. The position of two bottles were swapped. All rats were housed in a single cage throughout the test period and the weight of consumed solution of each rat (both 1.5% sucrose solution and plain water) was recorded. The sucrose preference rate was calculated (sucrose preference rate = consumption of sucrose solution/total consumption solution × 100%).

### Forced Swimming Test (FST)

The FST was used to assess behavioral despair. The test was carried out in a quiet environment. On the first day, rats were individually placed in a forced swimming bucket (a transparent tempered glass cylinder with a diameter of 21 cm and a height of 46 cm with a water depth of 30 cm, 25°C) and then taken out after 15 min of acclimatization, dried with a hair dryer, and placed back in the cage. On the second day at the same time, rats were placed into the forced swimming bucket again. All rats were tested and the time of immobility of each rat (the rat was passively floating with only the tail, slightly swinging with the paws to maintain body balance, and exposing the head to the surface of the water) was recorded within 5 min as an indicator. The swimming bucket was flushed, and water was changed after each trial to avoid affecting the behavior of the next rat.

### Enzyme-Linked Immunosorbent Assay (ELISA)

ELISA was used to detect the level of fecal corticosterone (CORT). The rats were housed singly, and feces of each rat were simultaneously collected for 24 h at the end of the 14th week (27–29). Feces fell freely through the bars of the wire cage and could be directly collected using the pan below each cage. The collected 24-h feces were homogenized in glacial acetic acid (2%). After being dried, ground, mixed, and large particles removed, feces were mixed with 80% methanol by high-speed vortex, followed by centrifugation at 2,500 × *g* for 10 min. The supernatant was mixed with EIA buffer as the test sample. The concentration of CORT in feces was measured using a Corticosterone EIA Kit (Cayman, Germany) following the manual instructions.

### Western Blot

The whole VTA tissue was dissected with coronal section rodent brain matrices (1 mm) according to the rat brain in stereotaxic coordinates and homogenized in ice-cold buffer (30). Western blot was performed as previously described. Anti-DRD2 antibody was purchased from Santa Cruz Biotechnology (San Diego, CA, USA). The horseradish peroxidase-conjugated anti-rabbit IgG antibody and anti-β-actin antibody were purchased from Sigma-Aldrich (St. Louis, MI, USA).

### Real-Time Reverse Transcription Quantitative PCR (RT-PCR)

RT-PCR was used to detect the DRD2 mRNA expression in the VTA. After the behavioral experiments, the rats were decapitated and the VTA tissues were stripped off quickly and transferred to liquid nitrogen. Total RNA was isolated from the dissected VTA tissues using Trizol reagent (Invitrogen, Carlsbad, CA, USA). RT-PCR was conducted as previously described (15). The primers for DRD2 were 5′-CTTGATAGTCAGCCTTGCTGTG-3′ and 5′-AGGGCACGTAGAATGAGACAAT-3′. The primers for β-actin were 5′-CACGATGGAGGGGCCGGACTCATC-3′ and 5′-TAAAGACCTCTATGCCAACACAGT-3′. The ΔΔCt method was used to calculate the results, and 2^−ΔΔCt^ represented the expression level of DRD2 mRNA. β-Actin mRNAs were used as an internal control.

### DNA Methylation Analysis

Genomic DNA was isolated from the VTA tissues by proteinase K/phenol-chloroform. DNA was quantified and then diluted to a working concentration of 10 ng/μl with TE buffer (Tris–EDTA). CpG islands locating in the promoter of DRD2 were selected for measurement according to the following criteria: (1) 200 bp minimum length; (2) 50% or higher GC content; (3) 0.60 or higher ratio of observed/expected dinucleotides CpG. One region from CpG islands (including 24 CpG sites) of DRD2 was selected and sequenced. Bisulfite amplicon sequencing PCR was used for quantitative methylation analysis by following a previously described protocol (31).

### Statistical Analysis

Data were presented as mean ± SEM and analyzed using the statistical software SPSS 21.0. One-way ANOVA and the least significant difference (LSD) method were used to compare differences among groups. Pearson correlation analysis was used to analyze the correlations between biomarkers and behavioral indexes. A *p* < 0.05 was considered as significant.

## Results

### Effect of MD on the Behaviors of Adulthood Rats

**Figure 1:**
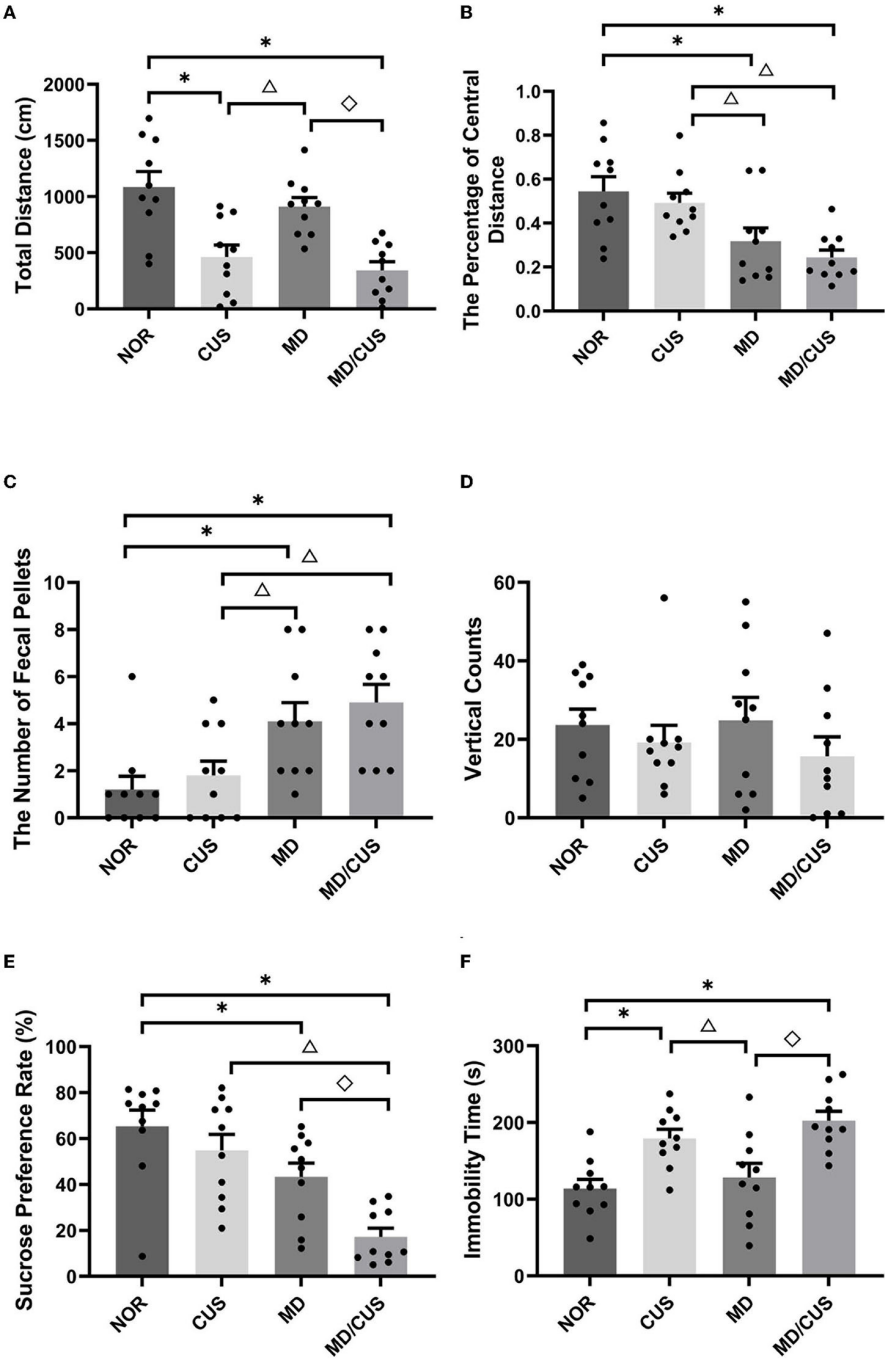
Effect of maternal deprivation on rats' behaviors in adulthood. **(A)** Total distance in the open field test. **(B)** The percentage of central distance in the open field test (the percentage of central distance = central distance/total distance). **(C)** The number of fecal pellets in the open field test. **(D)** Vertical counts in the open field test. **(E)** Sucrose preference rate in the sucrose preference test. **(F)** Immobility time in the forced swimming test. NOR: normal control (*n* = 10); CUS: chronic unpredictable stress (*n* = 10); MD: maternal deprivation (*n* = 10); MD/CUS: maternal deprivation plus chronic unpredictable stress (*n* = 10). ^*^p < 0.05 compared with NOR group. ^Δ^*p* < 0.05 compared with CUS group. ^♢^*p* < 0.05 compared with MD group.

OFT showed significant differences in the total distance (*F*_(3, 36)_ = 11.67, *p* < 0.001), the percentage of central distance (*F*_(3, 36)_ = 7.32, *p* = 0.001), and the number of fecal pellets (*F*_(3, 36)_ = 6.59, *p* = 0.001) among four groups. Specifically, CUS and MD/CUS rats had shorter total distance than NOR rats (post *p* < 0.05, Figure 1A). MD and MD/CUS rats showed a lower percentage of central distance (post *p* < 0.05, Figure 1B) and more feces (post *p* < 0.05, Figure 1C) than NOR rats. No significant difference in vertical counts was observed between four groups (*F*_(3, 36)_ = 0.742, *p* = 0.534; Figure 1D).

SPT showed a significant difference in the sucrose preference rate among four groups (*F*_(3, 36)_ = 11.51, *p* < 0.001). Specifically, MD and MD/CUS rats had significantly lower rate of sucrose preference than rats in the NOR group (post *p* < 0.05), whereas no significant difference in the sucrose preference rate was observed between CUS rats and NOR rats (post *p* > 0.05, Figure 1E).

FST showed significant differences in the immobility time among groups (*F*_(3, 36)_ = 9.07, *p* < 0.001). Specifically, CUS and MD/CUS rats showed significantly longer immobility time than NOR rats (post *p* < 0.05), whereas there was no significant difference in the immobility time between MD rats and NOR rats (post *p* > 0.05, Figure 1F).

### Effect of MD on Fecal CORT Concentration in Adult Rats

**Figure 2:**
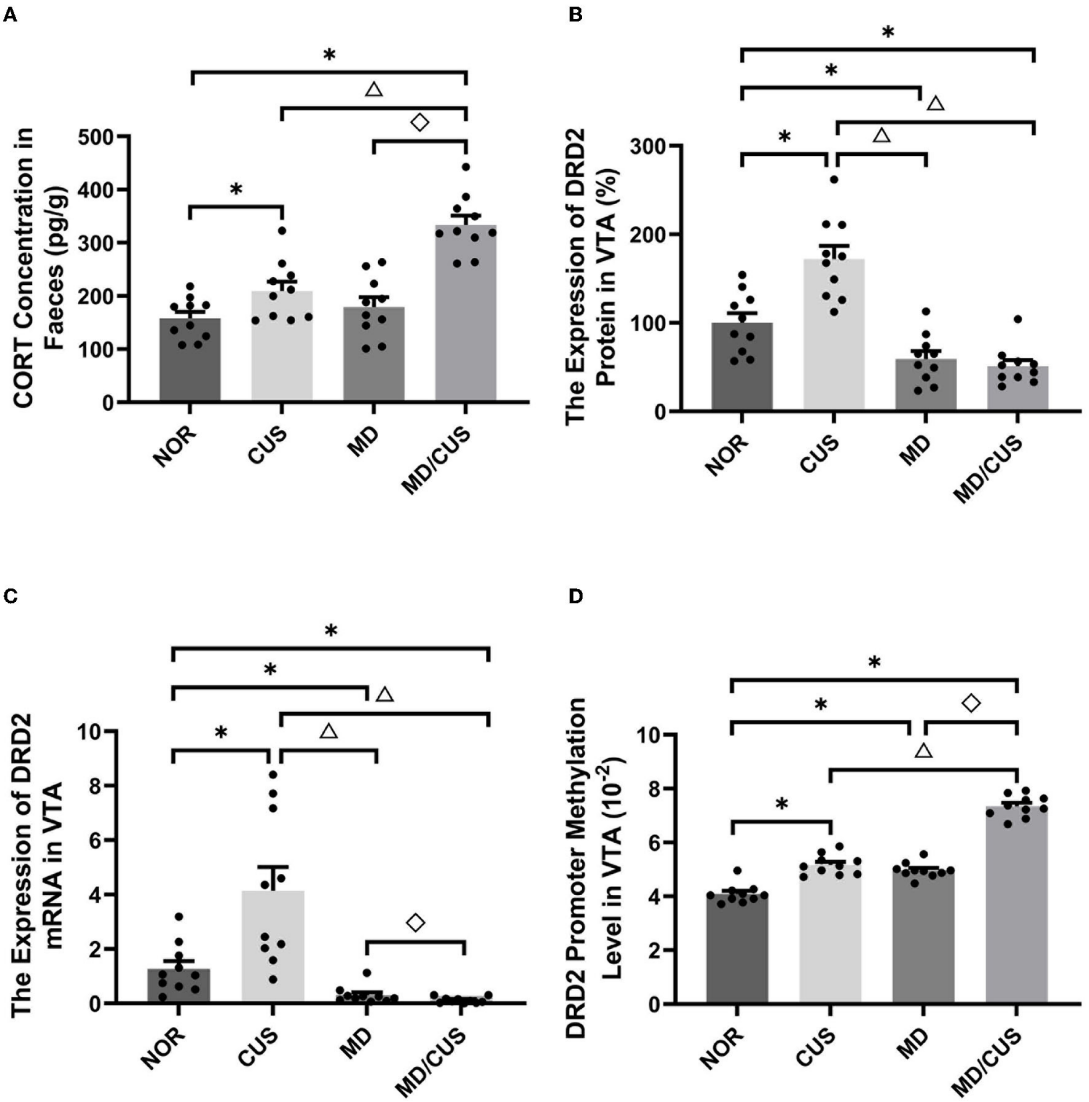
Effect of maternal deprivation on biomarker of rats in adulthood. **(A)** CORT concentration in feces. **(B)** The DRD2 protein level in the ventral tegmental area (VTA) tissues. **(C)** The DRD2 mRNA level in the VTA tissues. **(D)** The DRD2 promoter methylation level in the VTA tissues. NOR: normal control (*n* = 10); CUS: chronic unpredictable stress (*n* = 10); MD: maternal deprivation (*n* = 10); MD/CUS: maternal deprivation plus chronic unpredictable stress (*n* = 10). ^*^p < 0.05 compared with NOR group. ^Δ^ *p* < 0.05 compared with CUS group. ^♢^*p* < 0.05 compared with MD group.

Significant difference in fecal CORT concentration was observed among four groups (*F*_(3, 36)_ = 22.81, *p* < 0.001). Specifically, CUS and MD/CUS group showed a significantly higher CORT level in feces than NOR rats (post *p* < 0.05), whereas no significant difference in the fecal CORT concentration was observed between MD and NOR rats (post *p* > 0.05, Figure 2A).

### Effect of MD on the Level of DRD2 Protein, MRNA, and Methylation

Significant differences in DRD2 protein level in the VTA were observed among four groups (*F*_(3, 36)_ = 26.96, *p* < 0.001). Specifically, a significantly lower DRD2 protein level was observed in the VTA of MD and MD/CUS rats than NOR rats (post *p* < 0.05), whereas CUS rats showed significantly higher level of DRD2 protein in the VTA than NOR rats (post *p* < 0.05, Figure 2B).

The expression of DRD2 mRNA in the VTA was significantly different among four groups (*F*_(3, 36)_ = 16.09, *p* < 0.001). Specifically, MD and MD/CUS rats showed significantly lower expression of DRD2 mRNA than NOR rats (post *p* < 0.05), whereas CUS rats showed significantly higher expression of DRD2 mRNA than NOR rats (post *p* < 0.05, Figure 2C).

Significant differences in the level of DRD2 promoter methylation in the VTA were observed among groups (*F*_(3, 36)_ = 151.834, *p* < 0.001). Specifically, CUS, MD, and MD/CUS rats had a significantly higher level of DRD2 promoter methylation in the VTA than NOR rats (post *p* < 0.05). Moreover, MD/CUS rats had a significantly higher DRD2 promoter methylation level than MD and CUS rats (post *p* < 0.05, Figure 2D).

### The Correlations Between CORT, DRD2 Protein, DRD2 MRNA, DRD2 Methylation Levels, and Behavioral Indexes

**Figure 3:**
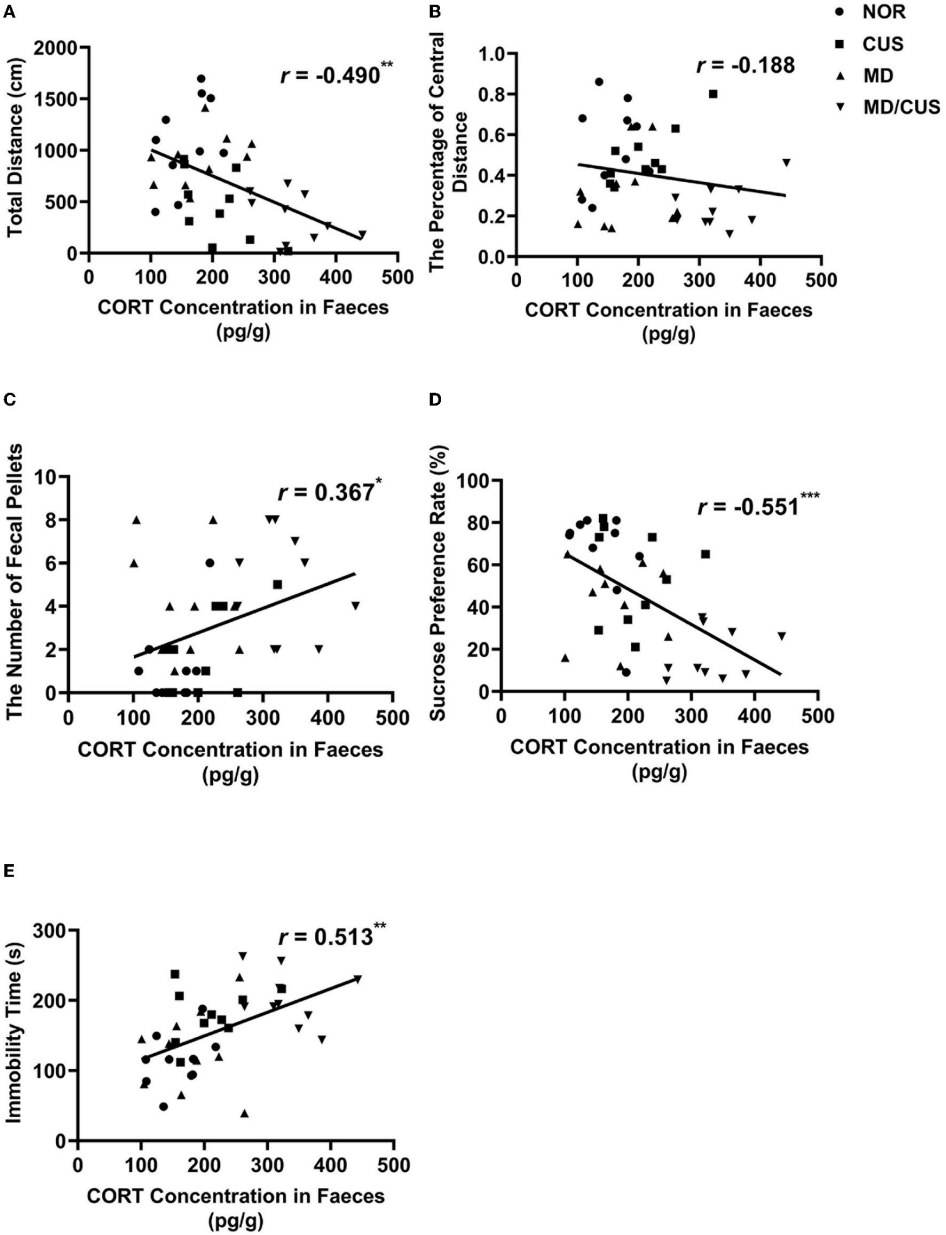
The correlations between CORT level in feces and behavioral indexes. **(A)** The correlation between CORT level in feces and the total distance in the open field test. **(B)** The correlation between CORT level in feces and the percentage of central distance in the open field test. **(C)** The correlation between CORT level in feces and the number of fecal pellets in the open field test. **(D)** The correlation between CORT level in feces and sucrose preference rate in the sucrose preference test. **(E)** The correlation between CORT level in feces and immobility time in the forced swimming test. Correlations were performed in all animals (*N* = 40). ^*^p < 0.05. ^**^p < 0.01. ^***^p < 0.001.

CORT level in feces was significantly correlated with the total distance (*r* = −0.490, *p* < 0.01, Figure 3A), the number of fecal pellets (*r* = 0.367, *p* < 0.05, Figure 3C), the sucrose preference rate (*r* = −0.551, *p* < 0.001, Figure 3D), and immobility time (*r* = 0.513, *p* < 0.01, Figure 3E). While there was no significant correlation with CORT level and the percentage of central distance (*r* = −0.188, *p* = 0.245, Figure 3B).

**Figure 4:**
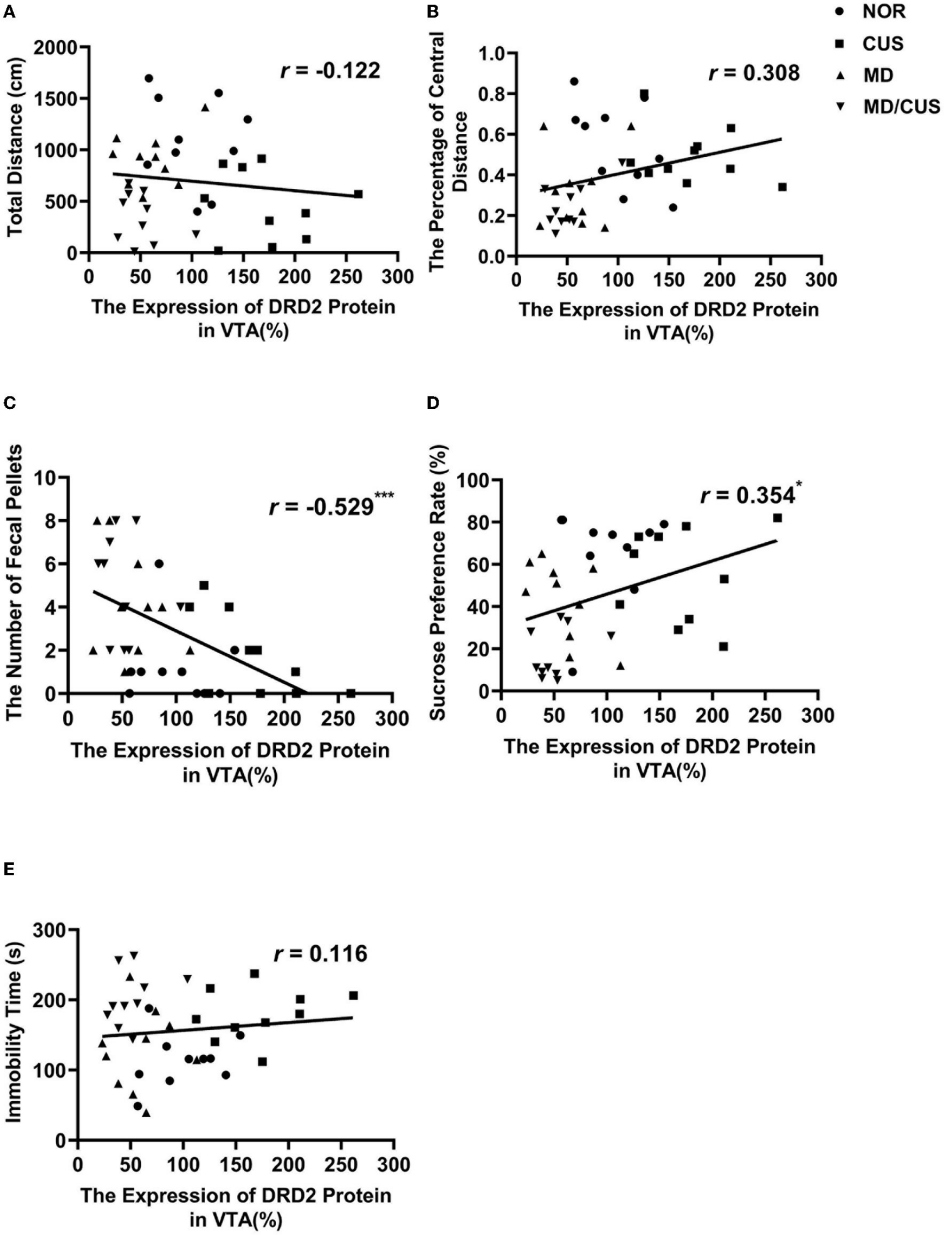
The correlations between DRD2 protein level in the VTA and behavioral indexes. **(A)** The correlation between DRD2 protein level in the VTA and the total distance in the open field test. **(B)** The correlation between DRD2 protein level in the VTA and the percentage of central distance in the open field test. **(C)** The correlation between DRD2 protein level in the VTA and the number of fecal pellets in the open field test. **(D)** The correlation between DRD2 protein level in the VTA and sucrose preference rate in the sucrose preference test. **(E)** The correlation between DRD2 protein level in the VTA and immobility time in the forced swimming test. Correlations were performed in all animals (*N* = 40). ^*^p < 0.05. ^**^p < 0.01. ^***^p < 0.001.

**Figure 5:**
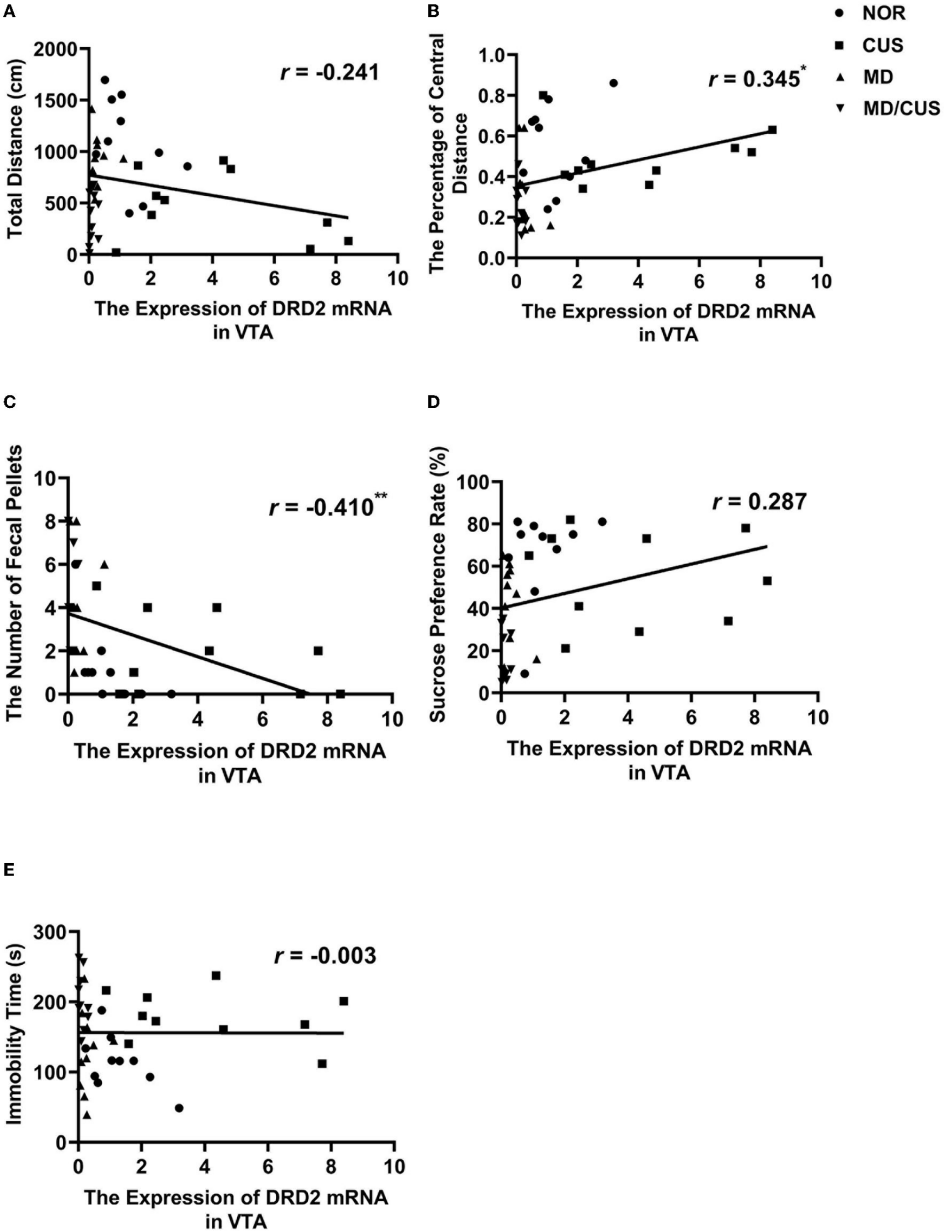
The correlations between DRD2 mRNA level in the VTA and behavioral indexes. **(A)** The correlation between DRD2 mRNA level in the VTA and the total distance in the open field test. **(B)** The correlation between DRD2 mRNA level in the VTA and the percentage of central distance in the open field test. **(C)** The correlation between DRD2 mRNA level in the VTA and the number of fecal pellets in the open field test. **(D)** The correlation between DRD2 mRNA level in the VTA and sucrose preference rate in the sucrose preference test. **(E)** The correlation between DRD2 mRNA level in the VTA and immobility time in the forced swimming test. Correlations were performed in all animals (*N* = 40). ^*^p < 0.05. ^**^p < 0.01. ^***^p < 0.001.

DRD2 protein level in VTA showed a significant correlation with the number of fecal pellets (*r* = −0.529, *p* < 0.001, Figure 4C) and the sucrose preference rate (*r* = 0.354, *p* < 0.05, Figure 4D). But the level of DRD2 protein were not significantly correlated with the total distance (*r* = −0.122, *p* = 0.454, Figure 4A), the percentage of central distance (*r* = 0.308, *p* = 0.053, Figure 4B), and the immobility time (*r* = 0.116, *p* = 0.475, Figure 4E). DRD2 mRNA level in VTA was significantly correlated with the percentage of central distance (*r* = 0.345, *p* < 0.05, Figure 5B) and the number of fecal pellets (*r* = −0.410, *p* < 0.01, Figure 5C). While DRD2 mRNA level were not significantly correlated with the total distance (*r* = −0.241, *p* = 0.134, Figure 5A), the sucrose preference rate (*r* = 0.287, *p* = 0.073, Figure 5D), and immobility time (*r* = −0.003, *p* = 0.984, Figure 5E).

**Figure 6:**
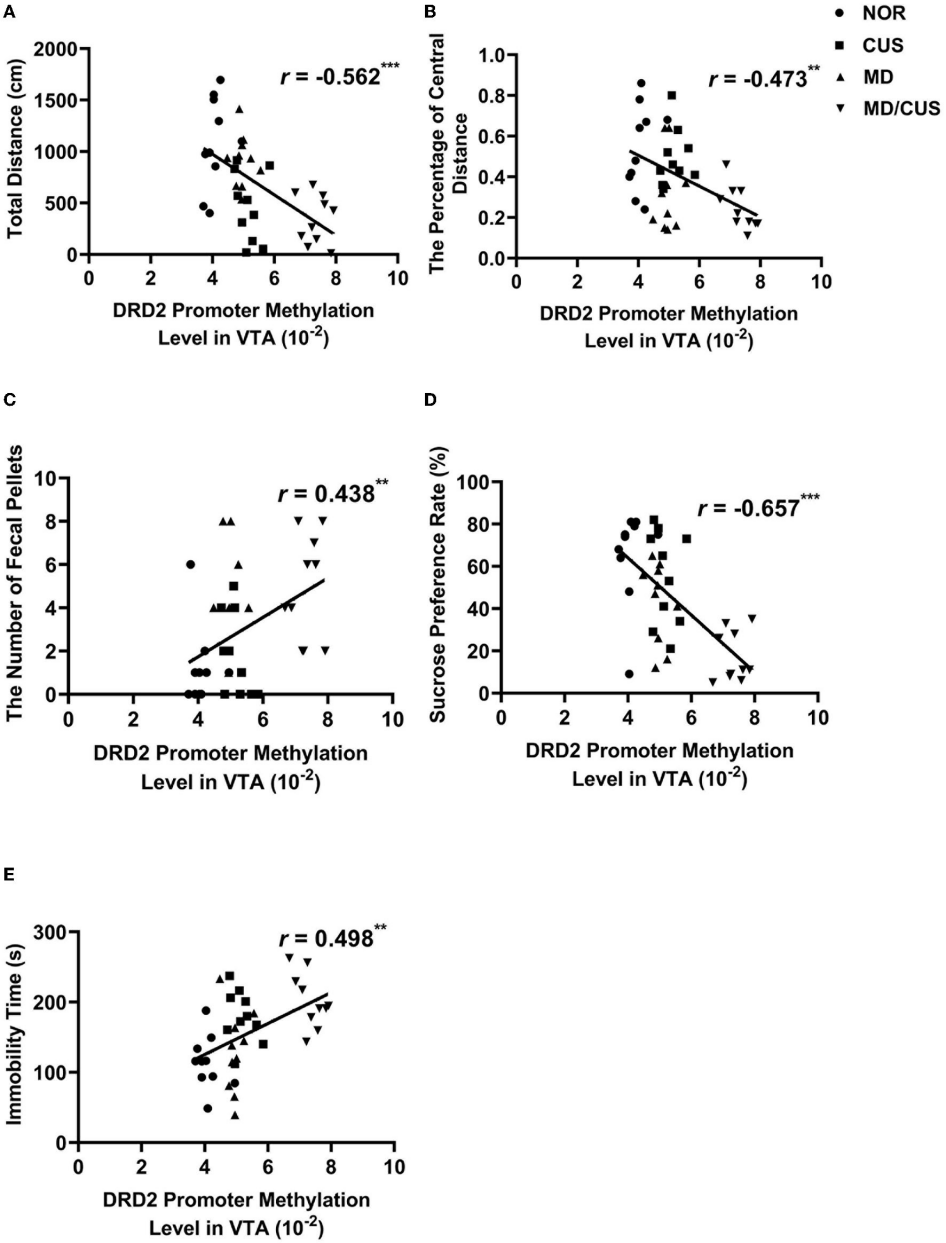
The correlations between DRD2 promoter methylation level in VTA and behavioral indexes. **(A)** The correlation between DRD2 promoter methylation level in VTA and the total distance in the open field test. **(B)** The correlation between DRD2 promoter methylation level in VTA and the percentage of central distance in the open field test. **(C)** The correlation between DRD2 promoter methylation level in VTA and the number of fecal pellets in the open field test. **(D)** The correlation between DRD2 promoter methylation level in VTA and sucrose preference rate in the sucrose preference test. **(E)** The correlation between DRD2 promoter methylation level in VTA and immobility time in the forced swimming test. Correlations were performed included all animals (*N* = 40). ^*^p < 0.05. ^**^p < 0.01. ^***^p < 0.001.

The level of DRD2 promoter methylation in VTA was significantly correlated with the total distance (*r* = −0.562, *p* < 0.001, Figure 6A), the percentage of central distance (*r* = −0.473, *p* < 0.01, Figure 6B), the number of fecal pellets (*r* = 0.438, *p* < 0.01, Figure 6C), the sucrose preference rate (*r* = −0.657, *p* < 0.001, Figure 6D), and immobility time (*r* = 0.498, *p* < 0.01, Figure 6E).

## Discussion

Maternal deprivation and chronic unpredictable stress paradigm are classical model for mimicking early life stress and stress experienced during adulthood, respectively. These stressors have been demonstrated to induce depression-like and anxiety-like behaviors in rodents (32–34). Our study revealed that maternal deprivation independently induces anxiety-like behavior (decreased percentage of central distance and increased number of fecal pellets in OFT), whereas chronic unpredictable stress alone induces despair-like behavior (increased immobility time in FST) and reduces locomotor activity (decreased total distance in OFT) in rats. It is worth noting that early life maternal deprivation plus chronic unpredictable stress can induce anhedonia-like behavior in adulthood rats, in addition to anxiety-like and despair-like behaviors (35). Previous studies demonstrated that individuals with high anxiety traits and anhedonia are more sensitive to stress-induced depression (36, 37). These findings suggest that early life maternal deprivation is generally regarded as a predictor of psychopathological vulnerability to depression in adulthood, but not a direct inducer of depression (38).

The dynamics of the hypothalamic–pituitary–adrenal (HPA) axis require balance of excitation and inhibition to adequately respond to stress and adapt to environmental demand (39). CORT has been identified as a biological marker of HPA axis activity, which relates to chronic stress and psychopathological vulnerability. Compared with traditional plasma samples, the quantification of CORT in feces offers the advantage of long-term monitoring of stress responses as a non-invasive method (40). In this study, chronic unpredictable stress and maternal deprivation plus chronic unpredictable stress both increased CORT level in feces. CORT level in feces was significantly correlated with the sucrose preference rate, immobility time, the total distance, and the number of fecal pellets. These findings are consistent with a previous study showing elevation of basal CORT levels and susceptibility to depression or anxiety after chronic stress (41, 42).

The dopaminergic system fully matures until young adulthood after development in the embryonic period (43). This prolonged development provides an extensive time window for the influence of early adverse events on vulnerability to psychopathology (43, 44). This study focused on the changes of DRD2 expression in the VTA in stress-induced depression and found that maternal deprivation reduced the expression of DRD2 gene. The reduction was more pronounced in maternal deprivation rats re-exposed to chronic unpredictable stress in adulthood. However, chronic unpredictable stress enhanced the expression of DRD2 gene in the VTA in adult rats without MD. It can also be seen that the correlation trends between DRD2 expression and behavioral changes induced by chronic unpredictable stress are different from other stresses, which could explain why the overall correlations between DRD2 expression and some behavioral indicators were not significant. Nevertheless, the influence of abnormal DRD2 expression cannot be ignored. In the VTA, DRD2 is a type of autoreceptor and expresses on the somatodentrites of dopaminergic neurons, which can inhibit the excitability of dopaminergic neurons by modulating the firing rate. The firing rate of dopaminergic neurons in the VTA was observed to increase in rodents that experienced chronic social defeated stress and chronic restraint stress (45–47), and decrease in chronic mild stress–induced rodents (48, 49), which are associated with increased vulnerability to depression (50). Authement et al.'s (51) study using a single 24-h episode of MD revealed that MD shifts spike timing-dependent plasticity toward long-term depression at GABAergic synapses onto VTA DA neurons and proposed that MD can promote DA dysregulation of VTA DA neurons through an epigenetic impairment of synaptic plasticity.

Previous studies proposed that epigenetic control of gene expression may be a bridge between early life stress and neurobehavioral outcomes in adulthood (52–54). For example, Godfrey et al.'s study suggested that epigenetic fine-tuning of the expression of genes can enhance the risk for depression in later life (55). This study showed that early life maternal deprivation increased the methylation of DRD2 promoter in the VTA. Indeed, the effect of maternal deprivation was more pronounced when rats were re-exposed to chronic unpredictable stress in adulthood. Furthermore, the level of DRD2 promoter methylation in the VTA was significantly correlated with the total distance, the percentage of central distance, the number of fecal pellets, the sucrose preference rate, and immobility time. Previous studies have demonstrated that the effect of maternal deprivation on DNA methylation of genes is associated with the susceptibility to depression (31, 56, 57). Collectively, long-term changes in the methylation of DRD2 promoter and its expression in the VTA induced by maternal deprivation may be related to the increased risk of developing depression later in life.

It should be known that many non-dopaminergic neurons exists in the VTA and D2 receptors also expressed on these non-dopaminergic cells (58, 59). For example, postsynaptic D2 receptors on the VTA GABAergic neurons can provide local inhibitory inputs onto dopaminergic neurons (60, 61) and knockdown of D2 receptors in GABAergic neurons can increase inhibitory tone (62). A previous study demonstrated that neuronal subpopulations are enriched in the VTA and other subpopulations of neurons may also express the DRD2 gene (63). Therefore, the expression of DRD2 in different types of neurons requires further investigations in the future.

We acknowledge that this study has several limitations. First, DRD2 expression on different subpopulations of neurons was not identified. In the future study, the cell-type specificity of DRD2 expression could be addressed using either immunohisochemistry or RNAscope staining. Second, what DNMT or DNMTs are responsible for the DRD2 gene methylation in the VTA is not identified. Thus, it is unknown whether DNMTs mediated DNA methylation in the different conditions. Previous studies demonstrated that quetiapine could reverse depressive-like behavior and reduce methylation induced by maternal deprivation through increasing DNMT activities in the hippocampus and NAc (64). Treatment of unstressed control rats with DNMT inhibitors recapitulated the effect of chronic unpredictable stress on decreased AMPAR expression and function in PFC. In contrast, overexpression of Dnmt3a in PFC of stressed animals prevented the loss of glutamatergic responses. Chronic stress-induced behavioral abnormalities could be partially attenuated by Dnmt3a expression in PFC (65). Third, this study only used male rats. Both sexes should be used to address whether there is a difference between males and females although most previous publications only used male animals. Fourth, in this study, only CpG islands locating in the promoter of DRD2 were selected for measurement. Although DNA methylation of the gene promotor is negatively correlated with gene expression, the differentially methylated CpG sites in the gene are positively and negatively correlated with gene expression, suggesting a bidirectional functional output on the enhancer and repressor regions of gene (66, 67). Data of this study are not able to answer why CUS and MD produce exhibited different effects on DRD2 expression. Whether differentially methylated CpG sites of the DRD2 gene, or other genes, and even other epigenetic mechanisms could account for the difference between CUS and MD should be addressed in future studies.

In conclusion, early life maternal deprivation increases vulnerability to stress-induced depressive-like behavior in adulthood. Enhanced DRD2 promoter methylation and subsequent downregulation of DRD2 expression in the ventral tegmental area may be involved in the increase in susceptibility to depression.

## Data Availability Statement

The original contributions presented in the study are included in the article/supplementary materials, further inquiries can be directed to the corresponding author.

## Ethics Statement

The animal study was reviewed and approved by the Animal Ethics Committee of the Second Xiangya Hospital, Central South University.

## Author Contributions

ZG, SL, and JW: performed experiments, data analysis, and prepared article. ZG, YZ, and XZ: study design, article revision, and final approval. SL and YZ: response to reviewers and article revision. All authors contributed to the article and approved the submitted version.

## Funding

This work was supported by National Natural Science Foundation of China (Nos. 81701333 and 81671341).

## Conflict of Interest

The authors declare that the research was conducted in the absence of any commercial or financial relationships that could be construed as a potential conflict of interest.

## Publisher's Note

All claims expressed in this article are solely those of the authors and do not necessarily represent those of their affiliated organizations, or those of the publisher, the editors and the reviewers. Any product that may be evaluated in this article, or claim that may be made by its manufacturer, is not guaranteed or endorsed by the publisher.
